# The Relationship Between Changes in Mindfulness and Subsequent Changes in Well-Being Following Psychedelic Use: Prospective Cohort Study

**DOI:** 10.2196/54632

**Published:** 2024-03-04

**Authors:** Grant Jones, Felipe Herrmann, Adam Bear, Robin Carhart-Harris, Hannes Kettner

**Affiliations:** 1 Harvard University Cambridge, MA United States; 2 University of California, San Francisco San Francisco, CA United States

**Keywords:** psychedelics, mindfulness, observational, web-based survey, psychedelic, meditation, mental health, anxiety, depression, survey, surveys, drug, drugs, substance use, hallucinogen, hallucinogens

## Abstract

This study demonstrates that changes in mindfulness predict subsequent changes in well-being in a data set including individuals who recently engaged in psychedelic use.

## Introduction

Psychedelics have been linked to improvements in depression, anxiety, and overall well-being in clinical trials and cross-sectional research [1-3]. Psychedelic use has also been linked to improvements in mindfulness [4,5], leading researchers to hypothesize that the link between psychedelic use and improvements in well-being may be driven in part by increased mindfulness [4,6,7]. Yet, there is a dearth of studies that directly explore this research domain.

This study uses a prospective data set of individuals who recently used psychedelics to examine, a priori, whether changes in mindfulness are linked to subsequent improvements in well-being, depression, and anxiety. Additionally, this study builds upon and addresses the core limitations of Mans et al [8], which found that psychedelic use was linked to improvements in mindfulness and well-being in the aforementioned data set. As Mans et al [8] did not control for major demographic and substance use confounds, include anxiety as an outcome in the analyses, nor assess how changes in mindfulness may potentially drive subsequent improvements in mental health, we aim to address these critical limitations within this study.

## Methods

### Overview

This study includes data that were collected in 2017 through a web-based platform called “Psychedelic Survey.” Haijen et al [9] provide further details on the data collection.

We used linear regression to assess whether changes in mindfulness from time 1 (baseline) to time 4 (2 weeks post psychedelic experience) were associated with changes in overall well-being, depression, and anxiety from time 1 to time 5 (4 weeks post psychedelic experience). We controlled for key demographic and substance use variables including age, gender, education level, and prior classic psychedelic use.

We used the Cognitive and Affective Mindfulness Scale–Revised to measure mindfulness, the Warwick-Edinburgh Mental Wellbeing Scale to measure overall well-being, the Quick Inventory of Depressive Symptomatology to measure depression, and the State-Trait Anxiety Inventory–6 (STAI6) to measure anxiety. As the STAI6 is commonly used to measure state (ie, momentary) anxiety, the scale was adapted to measure trait (ie, persisting) anxiety; these adaptations are included in Multimedia Appendix 1.

### Ethical Considerations

The study was approved by the Joint Research Compliance Office and the Imperial College Research Ethics Committee (ICREC reference 18IC4346). Participant data is anonymous, and participants were not compensated for their responses. All participants provided informed consent before participation.

## Results

Table 1 presents the demographics of our participants. Table 2 presents the results of our regression models. Changes in mindfulness (T1→T4) were associated with changes in overall well-being, depression, and anxiety (T1→T5). *R^2^* values ranged from 0.19 to 0.35, indicating moderate to strong effect sizes for our models. Multimedia Appendix 1 includes supplementary robustness analyses that assess the inverse relationship between well-being and mindfulness (ie, are changes in well-being from T1→T4 associated with changes in mindfulness from T1→T5?). Although these associations were significant, *R^2^* values for these models were lower than those for our main models assessing the relationship between mindfulness changes (T1→T4) and well-being changes (T1→T5; *R^2^* range 0.11-0.17). Thus, these additional results offer further evidence supporting the notion that mindfulness changes drove well-being changes in this study.

**Table 1:** Demographics of the sample.

Characteristic			Participants (N=163)
**Age (y)**			
	Median (IQR)	28 (23-38)	
	Range	16-71	
**Education, n (%)**			
	Left school before age 16 without qualifications	5 (3)	
	Some high school/GCSE^a^ level (in UK)	14 (9)	
	High school diploma/A-level education (in UK)	16 (10)	
	Some university (or equivalent)	26 (16)	
	Bachelor’s degree (or equivalent)	63 (39)	
	Post-graduate degree (e.g., masters or doctorate)	39 (24)	
**Gender, n (%)**			
	Male	113 (69)	
	Female	50 (31)	
**Prior psychedelic use, n (%)**			
	Never	14 (9)	
	Only once	11 (7)	
	2-5 times	43 (26)	
	6-10 times	23 (14)	
	11-20 times	23 (14)	
	21-50 times	30 (18)	
	51-100 times	7 (4)	
	More than 100 times	12 (7)	

^a^GCSE: General Certificate of Secondary Education.

**Table 2:** Results from three linear regression models assessing the relationship between how changes in mindfulness (independent variable [IV]; time 1→time 4) are associated with changes in overall well-being, depression, and anxiety (time 1→time 5; dependent variables [DV])^a^.

Characteristic	Overall well-being (T1→T5; DV)			Depression (T1→T5; DV)			Anxiety (T1→T5; DV)	
	Beta (95% CI)	*P* value	Beta (95% CI)		*P* value	Beta (95% CI)		*P* value
Mindfulness (T1→T4; IV)	0.71 (0.48 to 0.95)	<.001	–0.20 (–0.33 to –0.08)		.002	–0.35 (–0.44 to –0.26)		<.001
*R^2^*	0.291	N/A^b^	0.187		N/A	0.354		N/A
Adjusted *R^2^*	0.219	N/A	0.104		N/A	0.288		N/A

^a^Age, gender, education level, and prior psychedelic use are included in all models as covariates.

^b^N/A: not applicable.

## Discussion

Using a sample of individuals who recently used psychedelics, this study demonstrated that changes in mindfulness (T1→T4) were significantly associated with subsequent changes in mental well-being (T1→T5) [8]. Furthermore, models assessing the inverse associations between well-being and mindfulness (ie, assessing whether changes in well-being [T1→T4] predicted subsequent changes in mindfulness [T1→T5]) were weaker, strengthening the possibility that mindfulness drove improvements in well-being in this study.

Hill’s criteria for causation provide guidelines for evaluating the causal power of our results [10]. This study fulfills 7 of the 9 criteria: adequate strength of association, consistency with prior findings, specificity, plausibility, coherence (ie, biological plausibility), analogy (ie, comparability to related phenomena), and temporality (ie, the cause occurring before the effect). Additional research is needed to address the remaining criteria: biological gradient (ie, dose-response) and experimental evidence.

Limitations include the lack of a control group and the likelihood of bidirectional influence between changes in well-being and changes in mindfulness, both of which limit our ability to make definitive causal claims within this study. Another core limitation is our inability to control for important demographic factors in this study (eg, race/ethnicity, income, marital status). Future longitudinal studies and randomized trials can address these limitations. Overall, this study provides preliminary evidence that mindfulness may be a potential driver of the link between psychedelic use and salutary mental health outcomes.

## Appendix

Multimedia Appendix 1 Results from three linear regression models and the Adapted State-Trait Anxiety Inventory–6.

## Abbreviations

STAI6: State-Trait Anxiety Inventory–6

## Data Availability

Data sets are available upon request.
