# Supplementary material for: The Relationship Between Changes in Mindfulness and Subsequent Changes in Well-Being Following Psychedelic Use: Prospective Cohort Study
Source: JMIR Form Res. 2024 Mar 4;8:e54632. doi: 10.2196/54632 (PMC10949123; doi:10.2196/54632)
Supplement: Multimedia Appendix 1 [file formative_v8i1e54632_app1.docx]

**Supplementary Materials 1.** Results from three linear regression models assessing the relationships between how changes in overall wellbeing, depression, and anxiety (Time 1 → Time 4) (Independent Variables) are associated with changes in mindfulness (Time 1 → Time 5) (Dependent Variable). Age, gender, education level, and prior psychedelic use are included in all models as covariates.

|  | Mindfulness (T1→T5) (DV)^3^ | | | | | | | | |
| --- | --- | --- | --- | --- | --- | --- | --- | --- | --- |
| Characteristic | Beta | 95% CI^1^ | p-value | Beta | 95% CI^1^ | p-value | Beta | 95% CI^1^ | p-value |
| Wellbeing (T1 → T4) (IV)^2^ | 0.19 | 0.10, 0.29 | <0.001 |  |  |  |  |  |  |
| Depression (T1 → T4) (IV) |  |  |  | -0.25 | -0.44, -0.05 | 0.014 |  |  |  |
| Anxiety (T1 → T4) (IV) |  |  |  |  |  |  | -0.47 | -0.70, -0.23 | <0.001 |
| R² | 0.167 |  |  | 0.111 |  |  | 0.161 |  |  |
| Adjusted R² | 0.082 |  |  | 0.020 |  |  | 0.075 |  |  |
| ^1^CI = Confidence Interval; ^2^IV = Independent Variable; ^3^DV = Dependent Variable | | | | | | | | | |

**Supplementary Materials 2.** Adapted State-Trait Anxiety Inventory - 6

|  |  | Not at all | Somewhat | Moderately | Very much |
| --- | --- | --- | --- | --- | --- |
| 1. | Generally, I feel calm | 1 | 2 | 3 | 4 |
| 2. | Generally, I am tense | 1 | 2 | 3 | 4 |
| 3. | Generally, I feel upset | 1 | 2 | 3 | 4 |
| 4. | Generally, I am relaxed | 1 | 2 | 3 | 4 |
| 5. | Generally, I feel content | 1 | 2 | 3 | 4 |
| 6. | Generally, I am worried | 1 | 2 | 3 | 4 |

Please make sure that you have answered *all* the questions.
